# A Dermal Piercing Complicated by *Mycobacterium fortuitum*

**DOI:** 10.1155/2013/149829

**Published:** 2013-08-29

**Authors:** Trisha Patel, Leslie Scroggins-Markle, Brent Kelly

**Affiliations:** The University of Texas Medical Branch, 301 University Boulevard, Galveston, TX 77555, USA

## Abstract

*Background*. Dermal piercings have recently become a fashion symbol. Common complications include hypertrophic scarring, rejection, local infection, contact allergy, and traumatic tearing. We report a rare case of *Mycobacterium fortuitum* following a dermal piercing and discuss its medical implications and treatments. *Case*. A previously healthy 19-year-old woman presented complaining of erythema and edema at the site of a dermal piercing on the right fourth dorsal finger. She was treated with a 10-day course of trimethoprim-sulfamethoxazole and one course of cephalexin by her primary care physician with incomplete resolution. The patient stated that she had been swimming at a local water park daily. A punch biopsy around the dermal stud was performed, and cultures with sensitivities revealed *Mycobacterium fortuitum*. The patient was treated with clarithromycin and ciprofloxacin for two months receiving full resolution. *Discussion*. *Mycobacterium fortuitum* is an infrequent human pathogen. This organism is a Runyon group IV, rapidly growing nontuberculous mycobacteria, often found in water,soil, and dust. Treatment options vary due to the size of the lesion. Small lesions are typically excised, while larger lesions require treatment for 2–6 months with antibiotics. We recommend a high level of suspicion for atypical mycobacterial infections in a piercing resistant to other therapies.

## 1. Introduction

*Mycobacterium fortuitum *is a Runyon group IV, rapidly growing nontuberculous mycobacteria, often found in water (even municipal water systems), soil, and dust [1–5]. The portals of entry into humans include inhalation, mucosal, and via skin penetration [6]. Once it is has entered, it can cause respiratory infections, lymphadenitis, and skin/soft tissue infections, and in immunocompromised patients, it can lead to dissemination [5, 7]. Most common infections are cutaneous, usually associated with trauma or surgical procedures including liposuction, silicon injection, subcutaneous injections, acupuncture, and breast implants [3, 5, 7–10].

We report a case of *M. fortuitum *following a dermal piercing and discuss its medical implications and treatment. We also discuss other similar piercing infections caused by *M. fortuitum *and related mycobacteria. 

## 2. Case Report

A previously healthy 19-year-old Hispanic woman presented to our university-associated dermatology practice complaining of erythema, edema, and drainage at the site of a dermal piercing on the right fourth dorsal finger. The piercing was placed two months prior at a local piercing shop and became symptomatic approximately two weeks after she had received the piercing. She was treated with a 10-day course of trimethoprim-sulfamethoxazole and one course of cephalexin by her primary care physician with incomplete resolution. She did admit to swimming at a local water park daily. She was otherwise asymptomatic.

**Figure 1:**
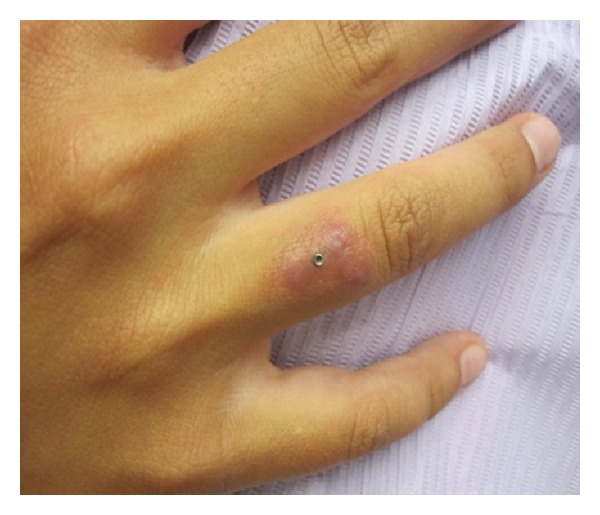
*M. fortuitum*. Clinical picture of dermal piercing site at the time of infection.

**Figure 2:**
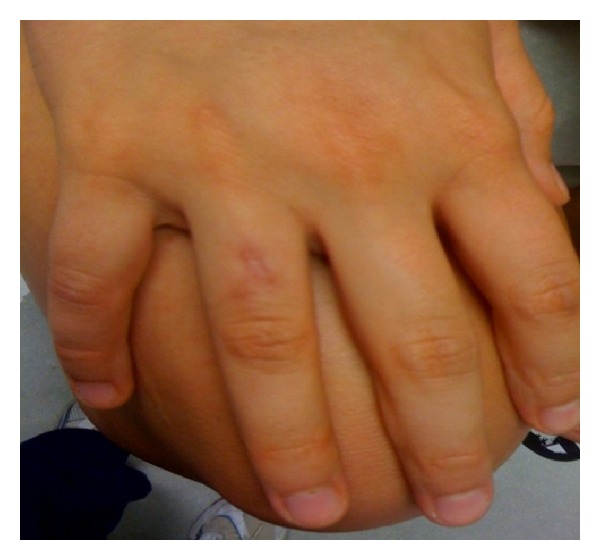
*M. fortuitum*. Clinical picture of dermal piercing site after full treatment; scar formation present from biopsy site.

On physical exam, the right fourth dorsal finger had 1.5 cm × 1 cm erythema and edema with tenderness to palpation. No drainage was appreciated (Figure 1). A 4 mm punch biopsy around the dermal stud was performed. The entire specimen was sent for tissue culture. At day four of culture, growth of atypical mycobacteria was observed, and by day 28, identification of growth and sensitivity was completed. Cultures grew 2+ *Mycobacterium fortuitum*, which was susceptible to amikacin, clarithromycin, ciprofloxacin, imipenem, and trimethoprim-sulfamethoxazole. Management included clarithromycin 500 mg by mouth twice daily and ciprofloxacin 500 mg by mouth once daily. After two months, full resolution of nodules was noted (Figure 2).

## 3. Discussion

Dermal piercings, also known as microdermal piercings, dermal anchoring, or “skin divers,” have recently become a fashion symbol. With only a stud visible on the skin surface, an “anchor” is placed just under the skin into the subcutaneous adipose and held in place by a metal plate. The anchor is placed into the layer of fat via an opening made with a dermal punch or large bore needle. The plate is then slid into place, and the stud is screwed on top. Common complications include keloid/hypertrophic scarring, rejection (less with titanium compared with other metals), local infection, endocarditis, communicable diseases, contact allergy, bleeding, migration, and traumatic tearing [9, 11]. 

*Mycobacterium fortuitum* is an infrequent human pathogen [1]. In culture, it can be detected within seven days along with other rapidly growing nontuberculous mycobacteria including *M. abscessus* and *M. chelonae* [1, 5, 12]. The clinical appearance of cutaneous *M. fortuitum *can vary, but most often appears as pustules, hyperkeratotic plaques, nodules with or without suppuration, a sporotrichoid pattern, or ulcers with draining sinuses [6]. Diagnosis is often made histologically with culture for confirmation. Histological appearance of *M. fortuitum* includes mixed acute and chronic granulomatous inflammation and is commonly presented with microabscess formation. Acid-fast organisms can be sparse and are not always seen [1]. 

Treatment options vary due to the size of the lesion. Small lesions can be excised [6]. Larger lesions must be treated according to sensitivities to antibiotics followed by excision. *M. fortuitum* is typically resistant to most antituberculous drugs, but has sensitivity to amikacin, clarithromycin, azithromycin, erythromycin, cefoxitin, doxycycline, and imipenem [1, 6]. It is recommended to use multiple drugs to reduce development of resistance. The recommended duration of treatment can vary but a duration of 2 to 6 months is usually needed. 

Other cases reporting of piercings associated with *M. fortuitum* include a 29-year-old female with bilateral breast abscesses mimicking carcinoma following bilateral nipple piercings, a 17-year-old female with bilateral breast abscesses following nipple piercings, and an 18-year-old female with a cheek abscess following a tragus piercing [12–14]. Other reported cases of atypical mycobacterial infected piercings include a 17-year-old female with nipple piercings who became infected with *M. abscessus*, a 22-year-old female with a navel piercing who became infected with *M. chelonae*, a 12-year-old female with an eyebrow piercing who became infected with *M. flavescens*, and a 35-year-old female with nipple piercings who became infected with *M. holsaticum*,* M. agri*, and *M. brumae* [11, 13].

## 4. Conclusion

We present a case of a dermal piercing of the finger complicated by *M. fortuitum* infection. It is possible that our patient was contaminated by piercing instruments or the jewelry, but it is also highly possible that she contracted the infection at the local swimming park or had prior skin inoculation. While we are unsure of the route of contamination, what is evident is that body piercings are continuing to become popular, readily available, and increasingly complex. As dermatologists, it is essential that we remain knowledgeable of what these piercings entail, as we are likely to see the complications of such piercings. Several recent case reports suggest that this as an emerging complication. A high index of suspicion should be maintained for atypical mycobacterial infection in a piercing resistant to other therapies; tissue cultures to evaluate for fungal and mycobacterial infections should be considered.
